# Extracellular Matrix Disorganization and Sarcolemmal Alterations in COL6-Related Myopathy Patients with New Variants of COL6 Genes

**DOI:** 10.3390/ijms24065551

**Published:** 2023-03-14

**Authors:** Simona Zanotti, Francesca Magri, Sabrina Salani, Laura Napoli, Michela Ripolone, Dario Ronchi, Francesco Fortunato, Patrizia Ciscato, Daniele Velardo, Maria Grazia D’Angelo, Francesca Gualandi, Vincenzo Nigro, Monica Sciacco, Stefania Corti, Giacomo Pietro Comi, Daniela Piga

**Affiliations:** 1 Neuromuscular and Rare Diseases Unit, Department of Neuroscience, Foundation IRCCS Ca’ Granda Ospedale Maggiore Policlinico, 20122 Milan, Italy; 2 Neurology Unit, Department of Neuroscience Foundation IRCCS Ca’ Granda Ospedale Maggiore Policlinico, 20122 Milan, Italy; 3 Dino Ferrari Centre, Department of Pathophysiology and Transplantation (DEPT), University of Milan, 20122 Milan, Italy; 4 IRCCS Eugenio Medea, Bosisio Parini, 23842 Lecco, Italy; 5 Medical Genetics Unit, Department of Medical Science, University of Ferrara, 44121 Ferrara, Italy; 6 Dipartimento di Medicina di Precisione, “Luigi Vanvitelli” University of Campania and Telethon Institute of Genetics and Medicine (TIGEM), 81100 Naples, Italy

**Keywords:** collagen type VI, extracellular matrix, electron microscopy, COL6-RM

## Abstract

Collagen VI is a heterotrimeric protein expressed in several tissues and involved in the maintenance of cell integrity. It localizes at the cell surface, creating a microfilamentous network that links the cytoskeleton to the extracellular matrix. The heterotrimer consists of three chains encoded by *COL6A1*, *COL6A2* and *COL6A3* genes. Recessive and dominant molecular defects cause two main disorders, the severe Ullrich congenital muscular dystrophy and the relatively mild and slowly progressive Bethlem myopathy. We analyzed the clinical aspects, pathological features and mutational spectrum of 15 COL6-mutated patients belonging to our cohort of muscular dystrophy probands. Patients presented a heterogeneous phenotype ranging from severe forms to mild adult-onset presentations. Molecular analysis by NGS detected 14 different pathogenic variants, three of them so far unreported. Two changes, localized in the triple-helical domain of COL6A1, were associated with a more severe phenotype. Histological, immunological and ultrastructural techniques were employed for the validation of the genetic variants; they documented the high variability in COL6 distribution and the extracellular matrix disorganization, highlighting the clinical heterogeneity of our cohort. The combined use of these different technologies is pivotal in the diagnosis of COL6 patients.

## 1. Introduction

Collagen VI is a non-fibrillar heterotrimeric protein expressed in the extracellular matrix (ECM) of connective tissue of several organs including skeletal muscle, skin, cornea, lung, blood vessels, intervertebral disks and joints. This complex localizes at the cell surface, links the cytoskeleton to the ECM and it is involved in cell anchoring and adhesion, maintenance of cell integrity and signal transduction. The heterotrimer consists of three main chains, alfa1, alfa2 and alfa3, which associate via their C-terminal domains and fold into triple helical monomers. These monomers align in an antiparallel manner to form dimers and tetramers representing the secreted form of collagen VI. Finally, in the extracellular space, tetramers associate end-to-end creating collagen VI microfibrils organized in a microfilamentous network [1]. These collagen chains are encoded by *COL6A1*, *COL6A2* and *COL6A3*. Molecular defects in one of these genes result in the assembly of mutated collagen VI filaments and determine compromised interactions with the other key components of ECM as collagen I, collagen II, fibronectin, glycosaminoglycans and proteoglycans [2]. This altered matrix organization affects the integrity of fibrillar network and compromises tissue homeostasis. Both recessive and dominant mutations in these genes cause a heterogeneous group of rare muscular disorders. The two main COL6 Related myopathies (COL6-RMs) are Ullrich congenital muscular dystrophy (UCMD, MIM 254090) and Bethlem myopathy (BM, MIM 158810) [3,4]. The Ullrich congenital muscular dystrophy is an early-onset severe and progressive disorder characterized by ambulation loss due to joint alterations, such as distal hyperlaxities and proximal contractures, scoliosis and respiratory failure. Bethlem myopathy is a relatively mild, slowly progressive disorder that usually becomes symptomatic with joint contractures in early childhood or adolescence, but it may also be present at birth. There is a phenotypic continuum between these border pathologies that distinguishes the intermediate COL6-RM (ICM), a new category characterized by a broad clinical spectrum and different degrees of weakness [5]. In addition, rarer phenotypes, such as myosclerosis myopathy and limb-girdle muscular dystrophy have been also reported [6,7].

The histological findings of the COL6-RM patient biopsies show muscle and connective tissue degeneration due to the absence or aberrant assembly of COL6 protein [8]. The muscle pathology includes fiber size disproportion, internal nuclei and prominent interstitial fibrosis with a scarcity of necrotic fibers [9]. 

Tissue fibrosis is a complex, not completely understood, progressive process characterized by the excessive deposition of ECM [10], that severely impairs tissue structure and function in diseases of various tissues including lung [11], liver [12], heart [13], skin and skeletal muscle [14,15]. Skeletal muscle fibrosis is a pathological hallmark of muscular dystrophies and myopathies representing a worsening trait in disease course [16].

The aim of our paper was to analyze clinical manifestations, pathological features and the mutational spectrum in a cohort of patients affected with COL6-related myopathy, referred to our Neuromuscular Unit. Different morphological approaches, including histological, immunological and ultrastructural techniques, allowed us to validate the new genetic variants and to elucidate the etiopathogenetic mechanisms underlying the ECM disorganization.

## 2. Results

### 2.1. Clinical Features 

We performed NGS/WES biomolecular analysis on a cohort of 53 genetically undiagnosed subjects with a history of progressive muscular weakness and myopathic changes at skeletal muscle biopsy and we identified 15 COL6-mutated subjects whose phenotype ranged from severe, childhood-onset forms with early loss of ambulation to milder adult-onset presentations. Five cases had no family history whereas the remaining 10 subjects were part of four different families for a total of nine involved families.

The mean age at the last evaluation was 46 ± 16 years. According to disease severity, the patients can be divided into two groups. Eight subjects presented a severe clinical phenotype with onset in childhood (4.8 ± 2.9 years of age) and severe progressive course with early tendon retractions and proximal weakness. Among them, two patients lost independent ambulation, respectively, at 5 and 10 years of age. Severe retractions were present in six of them. The other seven patients showed adulthood presentation with milder and slowly progressive neuromuscular involvement.

Considering the whole cohort, additional features, such as rigid spine, were present in four subjects, while three of them showed myopathic face with ptosis. Creatine phosphokinase (CPK) levels were generally mildly increased (2–10×), even if three patients presented normal values and one subject with congenital onset showed occasionally higher levels (60×). A respiratory involvement, characterized by a restrictive pattern, was present in six patients with a severe involvement in two of them. One patient showed obstructive sleep apnea syndrome. Cardiac function was normal in all patients. Electromyography was performed in four patients showing myopathic signs.

Detailed clinical data are summarized in Table 1.

### 2.2. Genetic Analysis

Molecular analysis by NGS and WES detected 14 different heterozygous nucleotide variants in the three main COL6 genes: three alterations were found in *COL6A1* (NM_001848), seven in *COL6A2* (NM_001849 as transcript reference sequence and NM_058174 for the transcript variant 2C2a) and four variants in *COL6A3* (NM_004369). Four molecular defects were novel and four had been reported recently (Table 2). The new variants were not found in the public databases (Ensemble, LOVD, dbSNP database). Molecular alterations were heterogeneous, including ten missense variants, one small in-frame deletion and three splice-site alterations. 

The frequency of the putative pathogenic missense variants was analyzed in the gnomAD database, finding a value of minimum allele frequency (MAF) less than 1%.

Two variants (Pts 1 and 4.1), localized in the Triple Helix (TH) domain, were involved in the monomer synthesis and in collagen assembly. The COL6A1 splicing mutations, c.958-2A>G in Pt 1 and c.930+189C>T in Pt 4.1, respectively, modified the conserved TH domain by the in-frame loss of three aminoacids in Pt 1 and by an in-frame inclusion of 24 aminoacids in Pt 4.1. In Pt 1, the pathogenetic role of this alteration was confirmed by the absence of the mutation in the parents. In addition the transcript analysis on muscle demonstrated the production of altered mRNA (r.958_966del), that predicted an in frame deletion at the protein level (p.Gly320_Lys322del).

The majority of variants (8 out of 14) were identified in different von Willebrand Factor A (vWFA) domains, that constitute the N- and C- globular portions of the protein and are important for the assembly and interaction of the collagens with other ECM components [17]. In particular, the alterations in COL6A2 and two missense variations in COL6A3 were localized in the C-globular region, that is essential for the first association of the three collagen VI α-chains, that wind together, producing a triple-helical monomer [18]. Instead, two variants were found in the COL6A3 N-globular region, which is important for the interaction of collagens with heparin and hyaluronan [19].

The new four genetic variants (*COL6A1:* c.628C>G; *COL6A2*: c.1806C>G, c.2145C>G and c.2738_2740del) were identified in Pts 6, 9.1, 7.1 and 8.1, respectively (Table 2). The segregation analysis of these genetic variants was performed only for the Pt 7; unfortunately, the unavailability of parents’ DNA samples prevented us from analyzing the other patients.

All the characteristics of each genetic variant are reported in Table 2.

**Table 2:** Genetic variants of patients.

Gene	Patient	Nucleotide Change	Protein Change	Protein Domain	ClinVar	Sift	PolyPhen 2	Mutation Tester	ACMG Classification	Reference
**COL6A1 NM_001848**										
	6	c.628C>G	p.(Arg210Gly)	vWFA1	_	Damaging	Probably damaging	Disease causing	Uncertain significance	New
	4.1	c.930+189C>T	p.Lys310_Gly311 insX [20]	THD	Pathogenic					[21]
	1	c.958-2A>G	p.(Gly320_Lys322del)	THD	Pathogenic	_	_	Disease causing	Pathogenic	rs1556425717
**COL6A2 NM_001849**										
	Family 9	c.1806C>G	p.(Cys602Trp)	Non-helical region	_	Damaging	Damaging	Disease causing	Uncertain significance	New
	Family 9	c.1832G>A	p.(Cys611Tyr)	Non-helical region	Probably damaging	Deleterious	Probably damaging	Disease causing	Uncertain significance	rs1375051583
	Family 7	c.2145C>G	p.(Ile715Met)	vWFA2	_	Damaging	Probably damaging	Disease causing	Uncertain significance	New
	Family 7	c.2192C>T	p.(Thr731Met)	vWFA2	Pathogenic	Deleterious	Probably damaging	Disease causing	Likely pathogenic	rs794727419 [22]
	Family 8	c.2423-2A>G	p.(Asp808_Thr820del)	Link	Pathogenic					[23]
	2	c.2503C>T	p.(Arg835Cys) ^1^	vWFA3	Uncertain significance	Deleterious	Possibly damaging	Disease causing	Likely benign	rs534856775
	Family 8	c.2738_2740del	p.(Ser913del)	vWFA3	_	_	_	Disease causing	Uncertain significance	New
**COL6A3 NM_004369**										
	2	c.1688A>G	p.(Asp563Gly)	vWFA3	Uncertain significance	Deleterious	Probably damaging	Disease causing	Uncertain significance	rs112913396 [24]
	5	c.2195C>T	p.(Thr732Met)	vWFA4	Uncertain significance	Deleterious	Possible damaging	Disease causing	Likely benign	rs370719148
	3	c.7928C>T	p.(Ala2643Val)	vWFA12	Uncertain significance	Deleterious	Possible damaging	Polymorphism	Benign	rs111595697
	3	c.8572G>A	p.(Val2858Ile)	Link	Uncertain significance	Tolerated	Benign	Polymorphism	Benign	rs111859552

^1^ RefSeq: NM_058174 for the transcript variant 2C2a.

### 2.3. Muscle Biopsy

To further characterize these patients, we performed a series of morphological, immunological and ultrastructural analyses on available muscle biopsies and cultured fibroblasts. 

**Figure 1:**
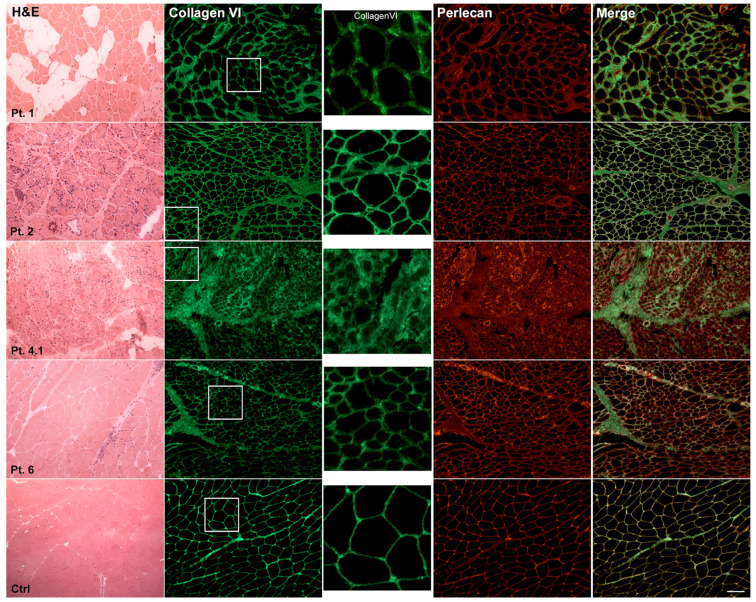
Histology and immunofluorescence on COL6-RM in pediatric patients. H&E staining showed marked fiber size variability, the presence of centronuclear fibers and a variable degree of fibrosis. Ctrl: aged-matched control muscle biopsy. Scale bar: 50 µm. Double fluorescent staining for collagen VI (green) and perlecan (red) showed an irregular membrane staining with a subtle signal reduction in Pts 1, 4.1 and 6 and a variable signal increase at perimysium in Pts 2 and 4.1. Pts 1 and 7.1 showed an enlargement between muscle fibers with collagen thickening. No patient showed a complete loss of collagen VI. The inset identifies the high magnification area. Scale bar: 50 µm.

**Figure 2:**
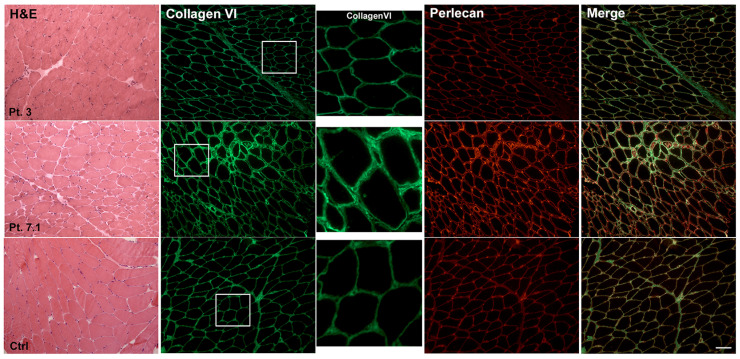
Histology and immunofluorescence on COL6-RM in adult patients. H&E staining shows marked fiber size variability and hypotrophic fibers, predominantly of type II (ATPase staining, data not shown) in Pt 7.1, and no significant changes in Pt 3. Ctrl: aged-matched control muscle biopsy. Scale bar: 50 µm. Double fluorescent staining for collagen VI (green) and perlecan (red) showed in Pt 3 a staining quite similar to control and a strong signal at the membrane and at the endomysium in Pt 7.1. The inset identifies the high magnification area. Scale bar: 50 µm.

Muscle biopsy from nine patients was available for histological analysis (Table 3). H&E staining revealed a marked fiber size variability and the presence of rare centronuclear fibers (Figure 1 and Figure 2). The quantification of fibrosis showed a slight to severe infiltration of fat and connective tissue. Pediatric patients showed a marked and significant increase in fibrotic tissue compared to age-matched controls. In detail, compared to age-matched controls (9.43 ± 2.14) fibrotic deposition was 27.72 ± 6.52 (*p* < 0.0001) in Pt 1, 39.73 ± 4.08 (*p* < 0.0001) in Pt 2.1, 39.92 ± 8.12 (*p* < 0.0001) in Pt 4 and 18.14 ± 5.28 (*p* < 0.0001) in Pt 6. Adult patients showed a significant fibrosis increase, though this was smaller than in pediatric patients. Indeed, compared to age-matched controls (9.11 ± 1.69), fibrosis was (12.91 ± 0.61, *p* = 0.0003) in Pt 5 and (14.31 ± 3.92, *p* < 0.0002) in Pt 7.1. No significant variation in connective tissue deposition was detected in Pt 3. Furthermore, in Pt 1, H&E staining showed a marked deposition of fat infiltration.

H&E staining showed marked fiber size variability, the presence of centronuclear fibers and a variable degree of fibrosis. Ctrl: aged-matched control muscle biopsy. Scale bar: 50 μm.

Double fluorescent staining for collagen VI (green) and perlecan (red) showed an irregular membrane staining with a subtle signal reduction in Pts 1, 4.1 and 6 and a variable signal increase at perimysium in Pts 2 and 4.1. Pts 1 and 7.1 showed an enlargement between muscle fibers with collagen thickening. No patient showed a complete loss of collagen VI. The inset identifies the high magnification area. Scale bar: 50 μm.

H&E staining shows marked fiber size variability and hypotrophic fibers, predominantly of type II (ATPase staining) in Pt 7.1, and no significant changes in Pt 3. Ctrl: aged-matched control muscle biopsy. Scale bar: 50 μm.

Double fluorescent staining for collagen VI (green) and perlecan (red) showed in Pt 3 a staining quite similar to control and a strong signal at the membrane and at the endomysium in Pt 7.1. The inset identifies the high magnification area. Scale bar: 50 μm.

### 2.4. Muscle Immunohistochemistry and Skin Fibroblasts Immunofluorescence 

Immunohistochemistry of collagen VI was performed on eight patients and four age-matched controls using mouse monoclonal antibody. No patient showed a complete loss of collagen VI; the most frequent observed alteration was an irregular membrane staining with a subtle signal reduction (Table 3 and Figure 1 and Figure 2). Double staining for collagen VI and perlecan, a large component of the basement membrane, was performed in order to investigate the exact/proper localization of collagen VI on the sarcolemma. The merged images showed a predominance of collagen VI signal in Pts 1 and 2, and a predominance of perlecan signal in Pts 4.1, 6 and 7.1. Pt 3 appeared quite similar to control. In Pts 1 and 7.1, the presence of an enlargement between muscle fibers was also observed as seen in UCMD patients with sarcolemma specific collagen VI deficiency (SSCD) (Figure 2) [20].

**Figure 3:**
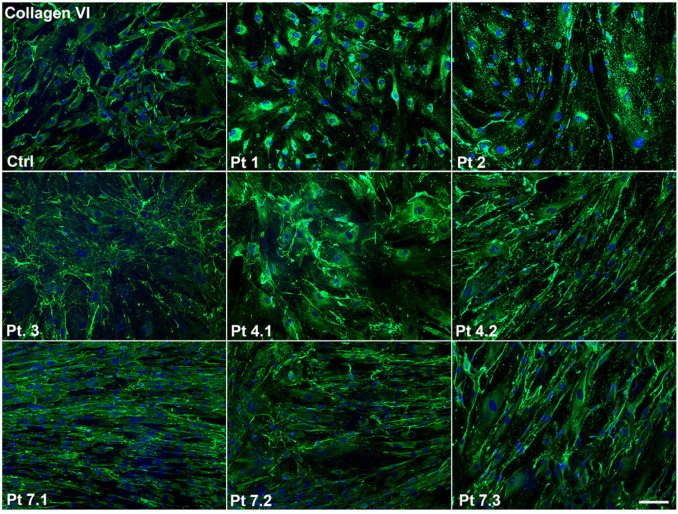
Immunofluorescent staining of Collagen VI on COL6-RM skin-derived fibroblasts. The staining shows variability in fluorescent signal distribution ranging from a complete collagen cellular retention (Pts 1 and 4.1) to altered extracellular deposition (Pt 2) and a variable collagen fibril arrangement (Pts 3 and 7.1). Nuclei were counterstained with DAPI. Ctrl: fibroblasts from normal skin biopsy. Scale bar: 50 µm.

The immunofluorescence analysis of collagen VI on skin fibroblasts isolated from eight patients and two controls, gave variable results, ranging from a complete collagen cellular retention of fluorescence inside the cytoplasm, as in Pt 1, to altered extracellular deposition, as in Pt 2. In this patient, a particular globular speckled pattern with fragmentation of the collagen VI signal was observed. Furthermore, fibroblasts immunofluorescent staining evidenced a different extracellular collagen VI fibril arrangement/orientation in the extracellular network: in the patients of Family 7, fibrils appeared parallelly oriented while in Pt 3 their deposition appeared as a “spider web” (Table 3 and Figure 3). 

In Pt 1 and Pt 4.1, which demonstrated differential effects on the assembly and secretion of collagen VI in muscle (Figure 1) versus skin cells (Figure 3), the more extensive treatment with ascorbic acid unmasked the release of the protein in the ECM (Supplementary Figure S1). In the same patients, we evaluated the deposition of collagen VI in not permeabilized fibroblasts and the production of other ECM components (fibronectin, collagen I and perlecan) (see Supplementary Figure S2). The merge between collagen VI and fibronectin demonstrated a similar colocalization of the proteins in the matrix, suggesting a normal interaction between collagen VI and fibronectin (Supplementary Figure S1). We observed a normal production of collagen I in Patient 4.1 and a slight reduction in Patient 1, whereas no clear immunofluorescence difference was observed in the expression of perlecan (Supplementary Figure S2).

The staining shows variability in fluorescent signal distribution ranging from a complete collagen cellular retention (Pts 1 and 4.1) to altered extracellular deposition (Pt 2) and a variable collagen fibril arrangement (Pts 3 and 7.1). Nuclei were counterstained with DAPI. Ctrl: fibroblasts from normal skin biopsy. Scale bar: 50 μm.

### 2.5. Western Blot

Western blot analysis performed on patients 1, 2, 3 and 4.1 and controls showed a reduction of collagen VI bands (a1/a2 chains 140 KDa) in Pt 1 and in Pt 2 and a normal signal for Pt 3 and 4.1. Western blot results are shown in Table 3 and Supplementary Figure S3.

### 2.6. Electron Microscopy Examination

**Figure 4:**
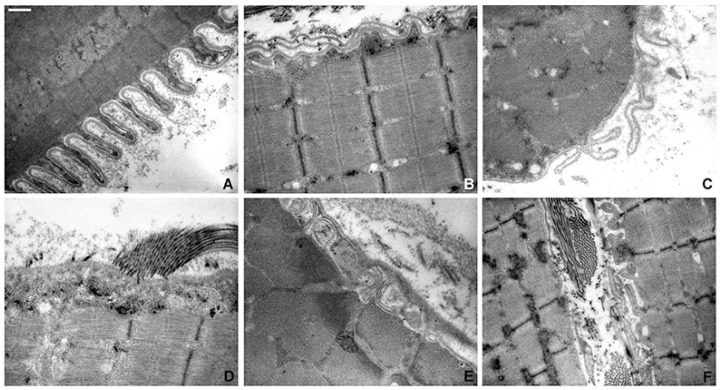
Ultrastructural investigation of muscle biopsies of COL6-RM. Representative ultrastructural images of skeletal muscle from Col6-RM patients. Papillary projections coursing perpendicularly (**A**) or horizontally (**F**) to the fiber surface. Basal lamina replication (**B**) or extroflection (**C**). Collagen fibrils closely localized at the basal lamina (**D**–**F**). Subsarcolemmal vacuoles containing membranous profiles (**E**). Scale bar: (**A**–**E**) 0.83 µm, (**F**) 1.43 µm.

Ultrastructural examination was performed on six patients (Table 3). A peculiar sarcolemma alteration characterized by microvilli-like projections was observed in four out of six subjects (Figure 4A). In a few cases, the basal lamina split away from the plasma membrane, forming folds in the extracellular space (Figure 4C,F) and sometimes the basal lamina was replicated (Figure 4B). The extracellular space contained an increased number of collagen fibrils that were closely applied to the external surface of the basal lamina (Figure 4D). Another peculiar alteration, observed in three biopsies (Pts 3, 5 and 6), was the presence of subsarcolemmal vacuoles, sometimes containing replicated membranes-like structures (Figure 4E). Less frequently, streaming of the Z line, increased glycogen content and rare apoptotic nuclei were observed.

## 3. Discussion

We describe the clinical, genetic and pathological data of 15 Italian patients affected with COL6-related myopathy, more specifically, five isolated cases and 10 subjects belonging to four families. On the basis of the recent classifications, seven subjects (Pts 1, 2, 4.1, 8.1, 8.2, 9.1 and 9.2) had a clinical diagnosis of Ullrich congenital muscular dystrophy, characterized by early onset and severe progressive course, whereas the other eight patients (Pts 3, 4.2, 5, 6, 7.1, 7.2, 7.3 and 7.4) could be classified as affected with Bethlem myopathy, i.e., with late onset and slowly progressive weakness [5,25].

The diagnostic complexity of COL6-related myopathies is such that the single genetic approach may not be sufficient to define the clinical picture, as the identified variants are often estimated by the software in a contradictory or, at the very least, uncertain way and sometimes disagree with the ACMG classification. 

Therefore, in support of the genetic investigation, it is essential to use other approaches, such as the morphological and ultrastructural studies on muscle biopsy or, if no muscle biopsy is available, on skin fibroblasts.

The pathogenicity of our genetic variants was well established by prediction tools and by parental segregation analysis only in two out of nine probands (Table 2). For the other subjects we applied a combination of diagnostic tools in order to define the pathogenicity of these molecular alterations, as discussed in the examples below.

In Pt 2, the genetic analysis identified two missense variants, one in COL6A2, in the 2C2a isoform, and one in COL6A3, the last one was already reported but in association with a COL6A1 mutation [24]. The genetic analysis identified the two variants but did not clarify the pathogenicity of the new association of variants in COL6A2 and COL6A3. In this regard, the morphological evaluation of the muscle biopsy turned out very informative, showing an increase in fibrosis and marked tissue structural alterations. Moreover, immunofluorescence staining detected an increase in COL6 perimysial signal and skin fibroblasts study showed a globular speckled pattern and fragmentation of collagen VI signal, similarly to what reported by Zhang Rui-Zhu in one patient carrying the E624K variant in COL6A2 [26]. This highly suggested the pathogenic role of this new variant association.

A different scenario occurred in Pt 5. In this patient the genetic analysis identified a new missense alteration. Querying prediction software showed a very variable response ranging from “deleterious” (SIFT) to “likely benign” (ACMG classification, version number 11.6.4). The segregation analysis was not performed for the lack of paternal DNA. Histochemical and immunofluorescence analyses showed no significant changes, but the ultrastructural analysis gave relevant clues to support the pathogenetic role of genetic variant. Indeed, pathological alterations such as microvilli-like projection, replication of basal lamina, subsarcolemmal vacuoles and collagen fibrils crossing the membrane were observed. 

In Pt 6, we identified a new genetic variant in the *COL6A1* gene for which it was impossible to evaluate parental segregation for pathogenicity validation. Muscle biopsy results were normal, and immunofluorescence showed a discrete increase of COL6 staining with a subtle reduction or absence of membrane staining in some fibers. Ultrastructural evaluation showed the presence of collagen fibrils through the membrane as well as vacuoles containing membranes-like structures and basal lamina extroflection and replication, thus confirming the pathological evidence seen at immunofluorescence. 

In Family 7, two genetic variants were identified in the *COL6A2* gene, one already described as pathogenic and the other never reported [22]. The segregation in cis of the two variants in all family members showed a dominant inheritance, therefore making a genotype-phenotype correlation quite difficult. 

We examined the skeletal muscle biopsy from the proband, and we performed fibroblast immunofluorescence on both the proband and the other family members. Despite the clinical heterogeneity among family members, we did not detect any differences in the fibroblast collagen VI signal, which suggests the necessity of further in vitro analysis.

Our results suggest the importance of ultrastructural insights on muscle biopsies from COL6-RM patients. In literature, few data about the ultrastructural alterations came from the studies in animal models [27,28]. Only a few papers reported ultrastructural alterations in muscle biopsies and muscle derived cells of COL6-RM patients. Tagliavini and colleagues (2014) described swollen mitochondria with paracrystalline inclusions and disorganized cristae [29], whereas other authors reported microfibril diameter size variation and a reduction of collagen VI-gold granules scattered around fibrillar collagens at the insertion into the basal lamina [2,30,31].

In our patients, sarcolemma, basal lamina and the subsarcolemmal region were prominently affected. We most frequently observed the formation of elongated microvilli-like projections born from the membranes (Figure 4A,F). In few cases, the basal lamina split away from the plasma membrane forming folds in the extracellular space (Figure 4C,F), and sometimes appeared thickened or replicated (Figure 4B); additionally, we observed subsarcolemmal vacuoles sometimes containing membrane-like elements (Figure 4E). Cenacchi and colleagues reported the presence of similarly elongated microvilli-like projections in muscle biopsies from LGMDR2 patients in association with a thickened basal lamina [32]. In addition, in CAV-3 patients, Kubisch and colleagues observed an increased surface area due to the formation of irregular folds and ridges or finger-like processes subtending a defective membrane repair [33].

We hypothesize that, in our patients, the incorporation of mutated collagen VI into the ECM altered the ability of collagen VI to bind other ECM macromolecules, i.e., decorin and biglycan, thus affecting the assembly and the structural integrity of the whole fibrillar network. This altered milieu may weaken the ECM mechanical support to muscle membrane. This instability could facilitate changes in the basal lamina, which can thicken or replicate, forming multiple layers in an attempt to protect the membrane, and favor the formation of microvilli-like projections. 

To summarize, the use of NGS methods has the potential to greatly improve our ability to make a firm diagnosis in a time- and cost-effective manner; however, given the high clinical heterogeneity and the variability in collagen expression, assembly and deposition, the complementary employment of different morphological techniques applied to patients’ skeletal muscle and skin tissues is pivotal not only to make the correct diagnosis, but also to understand the etiopathogenesis of COL6-related myopathies. 

## 4. Materials and Methods

Subjects were recruited at Fondazione I.R.C.C.S. Ca’ Granda Ospedale Maggiore Policlinico of Milan and IRCCS E. Medea Bosisio Parini.

This retrospective study was approved by the Institutional Review Board of the Fondazione I.R.C.C.S. Ca’ Granda Ospedale Maggiore Policlinico—University of Milan and written informed consent was obtained (and preserved in original) from all patients or their caregivers at the time of primary diagnostic procedures, with explicit consent to future uses for research purpose, according to the Declaration of Helsinki.

The “Telethon Bank of DNA, Nerve and Muscle Tissues” (no. GTF02008), located in the Department of Neurological Sciences, Fondazione I.R.C.C.S. Ca’ Granda Ospedale Maggiore Policlinico, Milan, Italy, was the source of the DNA samples used in this study.

Clinical data comprehensive of family history, detailed data of first symptoms, age of onset, presence of retraction and rate of disease progression were collected for all patients. Moreover, patients were followed over time with neurological, cardiac and pulmonary assessments.

Peripheral blood, skin biopsies, and muscle biopsy samples were obtained based on standard procedures.

Patient genomic DNAs were extracted from blood samples by standard procedures. Genetic analyses were performed over the years using Next Generation Sequence (NGS) protocols, based on different myopathy-related gene panels and by Whole Exome Sequencing (WES). Variants with minimal consequences on protein structure were rolled out and the remaining ones were filtered against Ensemble (https://www.ensembl.org/index.html; accessed on June 2022), LOVD (https://www.lovd.nl/; accessed on June 2022), dbSNP database (https://www.ncbi.nlm.nih.gov/snp/; accessed on June 2022) and allele frequency was evaluated using Exome Variant Server (https://evs.gs.washington.edu/EVS/; accessed on June 2022) and gnomAD database (https://gnomad.broadinstitute.org/; accessed on June 2022). We also evaluated the pathogenicity by in silico prediction programs: PolyPhen-2 (http://genetics.bwh.harvard.edu/pph2; accessed on June 2022), SIFT (https://sift.bii.a-star.edu.sg/; accessed on June 2022), Mutation Taster (https://www.mutationtaster.org/; accessed on July 2022) and ACMG classification (https://www.engenome.com/; accessed on September 2022). Sanger re-sequencing (3130 Genetic Analyzer, Applied Biosystems, Waltham, MA, USA) was used to confirm the rare variants in probands and available relatives. Genotype-phenotype associations were searched through the Pubmed, ClinVar (https://www.ncbi.nlm.nih.gov/clinvar/; accessed on September 2022), OMIM (https://www.omim.org/; accessed on June 2022), and Genetable database (https://www.musclegenetable.fr/; accessed on September 2022). 

For splicing alteration studies, total RNA was extracted from muscles using Eurogold RNA pure (Euroclone, Pero, Italy), retrotranscribed with a Transcriptor High Fidelity cDNA Synthesis kit (Roche, Basel, Switzerland). cDNA was analyzed by Sanger sequencing.

Skeletal muscle biopsies were performed at the Neuromuscular and Rare Diseases Unit and analyzed by both light and electron microscopy. Muscle sections from age-matched patients without any detectable muscle diseases were used as normal controls for immunohistochemistry and immunofluorescence analysis (all patients had signed written informed consent when they had undergone muscle biopsy).

Routine Haematoxylin and Eosin (H&E) histology was performed on 8 μm-thick cryostat muscle sections. On each section, four randomly, non-overlapping, selected fields were photographed at 20X magnification, using optical microscope Leica DC200 equipped with a camera and IM50 image analysis software (Leica Microsystems, Wetzlar, Germany). 

On H&E-stained muscle sections, the fibrotic area (connective and adipose tissue) was quantified by color subtraction using Leica Application Suite 4.9.0 and Fiji Image J software 2.1.0 (https://imagej.nih.gov/ij/download.html; accessed on May and June 2022) software. Briefly, on each H&E image, a color deconvolution was applied. The resulting image was then converted to a binary image to obtain fibers colored in white and connective and/or adipose tissue colored in black. Statistical analysis was performed using GraphPad Prism 5 software (GraphPad Software, LaJolla, CA, USA). Significant levels were set as *p* ≤ 0.001 (**) and *p* ≤ 0.05 (*). Fibrosis data were expressed as an area percentage (mean ± std. Dev.)

To process samples for a double immunofluorescence staining, skeletal muscle frozen sections (8 μm) were fixed with acetone for 1 min, washed three times with phosphate buffer saline (PBS) and blocked with 1% bovine serum albumin (BSA) in PBS for 60 min at room temperature (RT) and permeabilized with 0.1% Triton-X100 in PBS for 15 min at RT.

Sections were incubated, at the same times, with rat primary monoclonal antibody anti-heparan sulfate proteoglycan (Perlecan) (1:500 in PBS; MAB1948P from Merck, Kenilworth, NJ, USA), and with monoclonal antibody anti-collagen VI (1:200 in PBS; MAB1944, from Chemicon, Temecula, CA, USA), for 3 h at RT. Following three washing with PBS, sections were incubated with the mix of the two secondary antibodies, goat anti-rat Alexa-594 and goat anti-mouse Alexa-488 (both 1:500; Thermo Fisher, Waltham, MA, USA). Finally, after three washings with PBS, slides were mounted with anti-fading reagent Fluormount (Thermo Fisher). Images of single channel of immunolabelled sections were acquired using a Leica fluorescence microscope equipped with camera DFC420C (Leica). Merge images were obtained using Fiji Image J software.

Western blotting was performed on muscle homogenates from patients and controls. 

30 mg of samples were electrophoresed on 8% SDS-PAGE and transferred onto nitrocellulose membranes. Membranes were probed with antibody to collagen VI (1:3000 rabbit polyclonal; Fitzgerald 70R-CR009x) and actin (1:1000 rabbit polyclonal; Sigma-Aldrich, Burlington, MA, USA). Actin was used as an indicator of protein loading. Blocked membranes were then incubated in HPR-conjugated secondary antibody (1:3000; Dako, Glostrup, Denmark) and then detected with the ECL chemiluminescence reagent (Amersham Biosciences, GE Healthcare, Little Chalfont, UK).

Western blot bands had been quantified by using Image J 1.46r software. Densitometric data of collagen VI were normalized to actin and expressed as a percentage of the control.

For ultrastructural examination, a small part of a muscle sample was fixed in 2.5% glutaraldehyde (pH 7.4), post fixed in 2% osmium tetroxide and then, after dehydration in a graded series of ethanol, embedded in Epon’s resin. Finally, ultrathin sections were stained with lead citrate and uranyl acetate and examined with a Zeiss EM109 transmission electron microscope.

Skin biopsies from normal controls and patients were performed in order to obtain dermal fibroblast cultures, which were maintained in Dulbecco’s modified Eagle medium (Gibco) with 10% fetal bovine serum (Gibco) and 1% penicillin/streptomycin (Gibco) in 5% CO_2_ at 37 °C. For immunofluorescence staining, skin fibroblasts cultures were treated with 50 ug/mL sodium L-ascorbate (Sigma) for 3 days after confluence at 80%. Cells were fixed in 4% paraformaldehyde for 10 min at 4 °C, washed three times with PBS 1X, blocked and permeabilized with 10% BSA plus 0.3% Triton in PBS for 60 min at RT. Fibroblasts were incubated over night at 4 °C with a rabbit primary polyclonal antibody anti-collagen VI (1:250 in 3% BSA; Fitzgerald 70R-CR009x). After three washings with PBS, cells were incubated with a secondary antibody, goat anti-rabbit Alexa-488 (1:1000 in 3% BSA; Thermo Fisher, Waltham, MA, USA) and DAPI (4′,6-diamidino-2-phenylindole) was applied for counterstaining nuclei. 

Only skin fibroblasts cultures of Pt 1 and 4.1 were additionally treated with L-ascorbate for 15 days after confluence. Cells were fixed in paraformaldehyde and permeabilized/not permeabilized with 10% BSA plus 0.3% Triton in PBS for 60 min at RT. For the staining, samples were incubated overnight with different primary antibodies: rabbit polyclonal antibody anti-collagen VI (1:250; Fitzgerald 70R-CR009x), rabbit polyclonal antibody anti-collagen I (1:100; Abcam ab34710), rat monoclonal antibody anti-heparan sulfate proteoglycan (Perlecan) (1:100; Merck MAB1948P) and mouse monoclonal antibody anti-fibronectin (1:100; Merck F7387) used for the double-staining. As secondary antibodies we used: goat anti-rabbit Alexa-488, goat anti-rabbit Alexa-594, goat anti-rat Alexa-594 and goat anti-mouse Alexa-568 (1:1000; Thermo Fisher, Waltham, MA, USA). Nuclei were counterstained with DAPI.

After three washing with PBS, slides were mounted with Fluor-Save reagent (Sigma) and images of immunolabelled fibroblasts were acquired using a Leica fluorescence microscope equipped with camera DFC420C (Leica). Merge images were obtained using Fiji Image J software.

## Figures and Tables

**Table 1:** Demographic and clinical characteristics.

Patient	Sex	Age (Years)	Age of Onset (Years)	First Symptoms	CLINICAL CLUES	Retractions	CPK (U/L)	Respiratory Difficulty	Others
**1**	F	30	4	Motor development delay Hypotonia at birth	Proximal and distal weakness (4 limbs) Loss of independent ambulation (10 years)	Yes (Multiple surgery)	300	Mild restrictive	Cheloids, scoliosis
**2**	F	26	4	Weakness of the lower limbs	Proximal and distal weakness (4 limbs)	Yes	2568–12,518	Mild restrictive	_
**3**	F	34	2	Fingers flexors contractures (2 years)	Proximal and distal weakness (4 limbs)	Yes (Multiple surgery)	Normal	No	Rigid spine, ptosis, bilateral cataract
**4.1**	M	16	1.2	Mild proximal weakness motor milestones delay	Proximal weakness (4 limbs), loss of independent ambulation (5 years)	Yes	Normal-258	Mild restrictive	Ptosis, mild scoliosis, hyperlaxity
**4.2**	M	48	_	Mild right ptosis	Mild proximal weakness at lower limbs	No	2500	Mild restrictive	_
**5**	F	72	49	Proximal weakness	_	_	3000	_	_
**6**	F	27	_	_	_	_	_	_	_
**7.1**	M	57	_	Proximal weakness	Proximal > distal weakness (upper > lower limbs)	Yes	_	Obstructive sleep apnoea syndrome	_
**7.2**	M	47	_	Mialgia and cramps	No motor impairment up to 43 years	Yes	700–800	No	_
**7.3**	F	58	_	Mild CPK increase	Proximal and distal weakness lower limbs	_	Mild increase	No	Rigid trunk
**7.4**	F	61	_	Mild CPK increase	Mild proximal weakness	_	_	_	_
**8.1**	M	59	Early childhood	Mild proximal weakness	Proximal and distal weakness (4 limbs)	No	_	No	Dysphagia
**8.2**	F	68	Early childhood	Weakness and fatigue	Proximal and distal weakness (4 limbs)	No	Normal	No	_
**9.1**	M	56	4	Weakness lower limbs	Proximal and distal weakness (4 limbs)	Yes (moderate)	203–523	Severe restrictive	Rigid spine
**9.2**	M	41	10	Weakness lower limbs	Proximal and distal weakness (4 limbs)	Yes (moderate)	190–425	Severe restrictive	Rigid spine

**Table 3:** Morphological, immunological and ultrastructural characteristics.

Patient	Fiber Size Variability	Fiber Type Distribution	Centronuclear Fibers	Fibrosis (%Area)	Muscle Tissue IHC COL6	Western Blot COL6	Skin Fibroblast IF COL6	Ultrastructural Analysis M.E.
**1**	Marked	Correct typological differentiation and topographic distribution	Rare	27.72 ± 6.52	Widespread increase/some fibers with a subtle reduction/absence membrane staining	Slight reduction	Retention	Microvilli-like projections of membrane/Collagen fibrils through the membrane/Increase glycogen content
**2**	Marked variability	_	No	39.73 ± 4.08	Increase at perimysium Normal at membrane	Reduction	Partial retention/globular speckles	_
**3**	Mild	Correct typological differentiation Hypotrophic fibers predominantly of type II	No	11.48 ± 2.71	Normal	Normal	Patchy distribution	Subsarcolemmal vacuoles, sometimes containing replicated membranes-like
**4.1**	Marked	Correct typological differentiation Hypotrophic fibers of both types	Rare	39.92 ± 8.12	Marked increase at perimysium Some fibers with irregular sarcolemma staining	Normal	Retention	Microvilli-like projections of membrane/Rare streaming of Z line/Rare dilation of sarcoplasmic reticulum
**4.2**	Normal	Correct typological differentiation and topographic distribution	Rare	12.82 ± 2.86	Some fibers with a subtle reduction Absence membrane staining	_	Weak distribution	_
**5**	Discrete	Hypotrophic fibers of both types	Rare	12.91 ± 0.61	_	_	_	Sarcolemma extroflection/Basal lamina replication/Collagen fibrils through the membrane/Subsarcolemmal vacuoles
**6**	Marked	Hypotrophic fibers predominantly of type I	No	18.13 ± 5.28	Discrete increase Some fibers with a subtle reduction/absence membrane staining	_	_	Collagen fibrils through the membrane Subsarcolemmal vacuoles, containing membranes-like Basal lamina extroflection and replication Rare streaming of Z line
**7.1**	Marked	Hypotrophic fibers predominantly of type II	Rare	14.30 ± 3.99	Strong labelling of the basement membrane and endomysium	_	Partial retention, patchy distribution	Collagen fibrils go through the membrane Increase glycogen content
**7.2**	_	_	_	_	_	_	Partial retention patchy distribution	_
**7.3**	_	_	_	_	_	_	Partial retention patchy distribution	_
**9.2**	Discrete	_	Slightly increase	14.83 ± 1.09	Some fibers with a subtle reduction Absence membrane staining	_	_	_

## Data Availability

The data presented in this study are available on request from the corresponding author.

## Supplementary Materials

The following supporting information can be downloaded at: https://www.mdpi.com/article/10.3390/ijms24065551/s1.
