# Supplementary material for: Extracellular Matrix Disorganization and Sarcolemmal Alterations in COL6-Related Myopathy Patients with New Variants of COL6 Genes
Source: Int J Mol Sci. 2023 Mar 14;24(6):5551. doi: 10.3390/ijms24065551 (PMC10059973; doi:10.3390/ijms24065551)
Supplement: Supplementary file 1 [file ijms-24-05551-s001.zip › Supplementary.pdf]

Supplementary legends

Supplementary Figure S1: Immunofluorescent staining of Collagen VI and Fibronectin on COL6-RM skin-derived fibroblasts. The fluorescent signal after 15 days treatment with sodium L-ascorbate unmasked a marked deposition of collagen VI in the ECM and a similar pattern was observed in the fibronectin staining. The merge of collagen VI and fibronectin demonstrated a normal interaction between these proteins. We included two controls with different degree of confluence to better compare them with patients' fibroblasts. Nuclei were counterstained with DAPI. Scale bar: 50 μm.

Supplementary Figure S2: Immunofluorescent staining of Collagen I, Perlecan and Collagen VI. The fluorescent signal evidenced a normal production of collagen I in Patient 4.1 and a slight reduction in Patient 1, whereas no clear immunofluorescent difference was observed in the expression of perlecan. Comparison of collagen VI signal in patients and controls with and without Triton permeabilization showed that the extracellular signal of the protein was more evident in non-permeabilized cells in both control and patients' cell lines. In permeabilized cells a residual cytoplasmic signal was still detected. We included two controls with different degree of confluence to better compare them with patients' fibroblasts. Nuclei were counterstained with DAPI. Scale bar: 50 μm.

Supplementary Figure S3: Western Blot of Collagen VI. Western Blot showed a reduction of α1/ α2 chains (140 KDa band) of Collagen VI in selected patients Pt 1, Pt 2 compared to controls. A non-specific band running at 200 KDa was also detected (asterisk), probably corresponding to the myosin HC. Actin (45 KDa band) was used as protein loading control.
