# Supplementary figures and images for: Extracellular Matrix Disorganization and Sarcolemmal Alterations in COL6-Related Myopathy Patients with New Variants of COL6 Genes
Source: Int J Mol Sci. 2023 Mar 14;24(6):5551. doi: 10.3390/ijms24065551 (PMC10059973; doi:10.3390/ijms24065551)

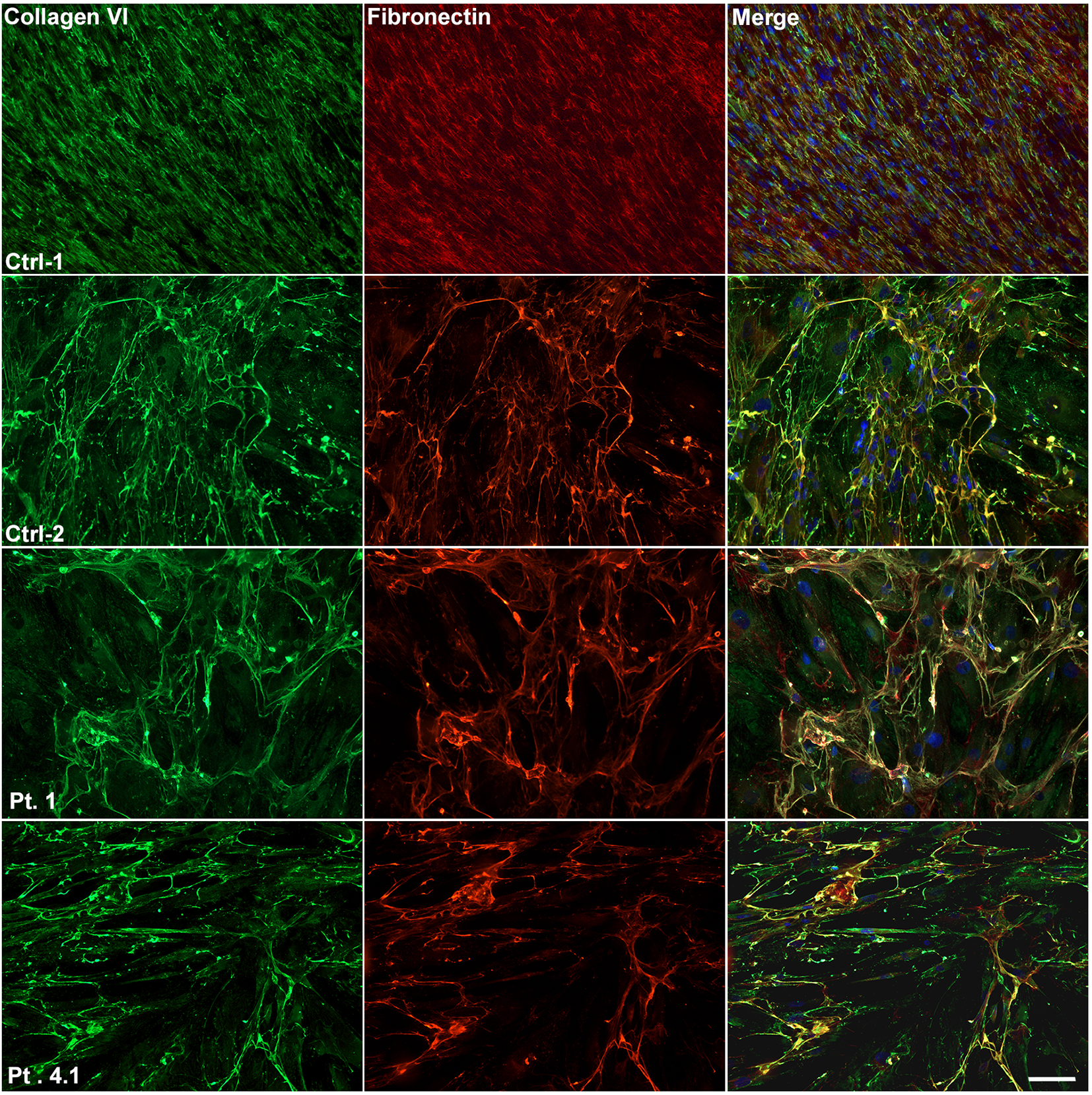

Supplement: Supplementary file 1 [file ijms-24-05551-s001.zip › Figure S1 supplementary.tif]

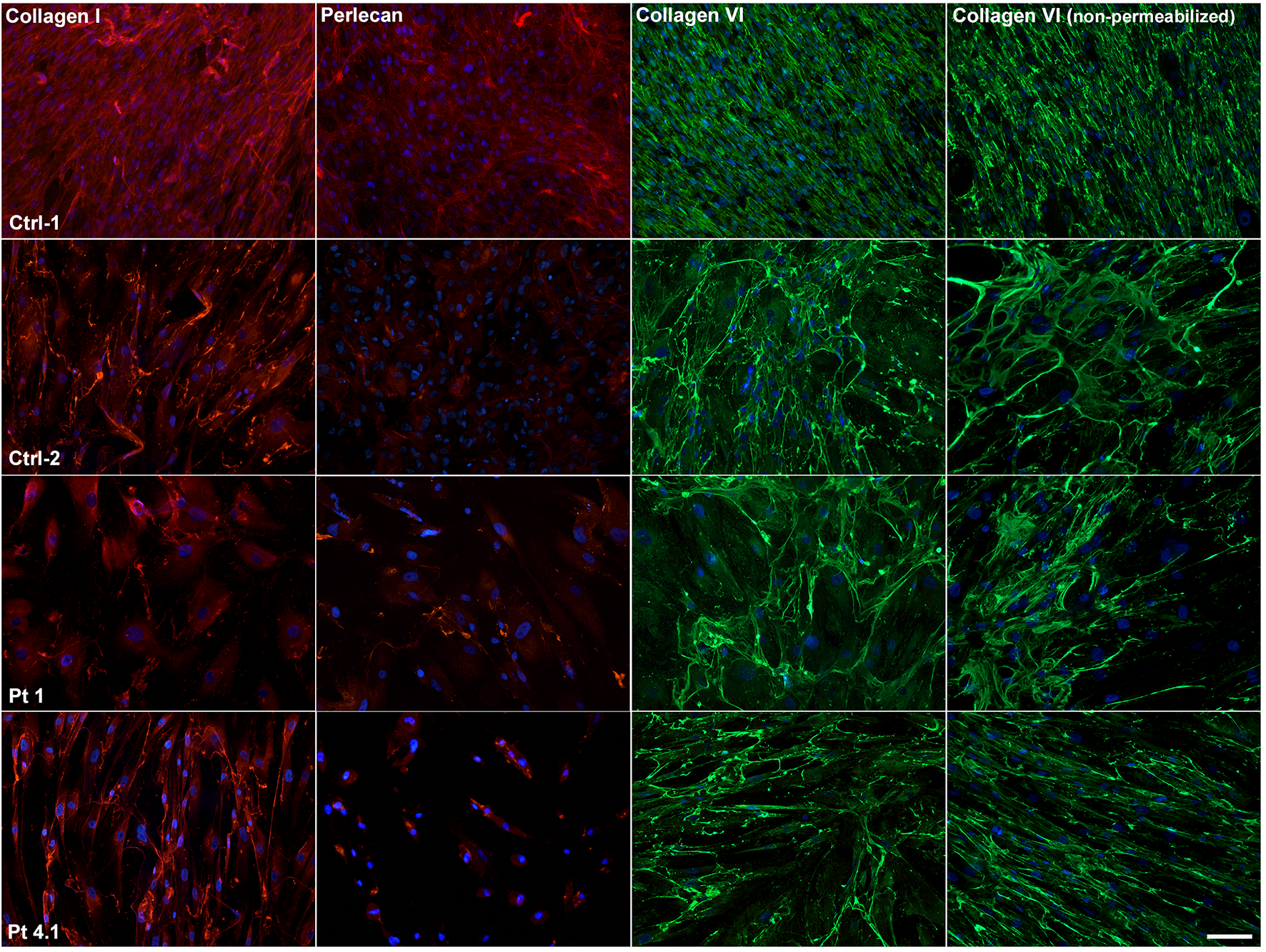

Supplement: Supplementary file 1 [file ijms-24-05551-s001.zip › Figure S2 supplementary.tif]

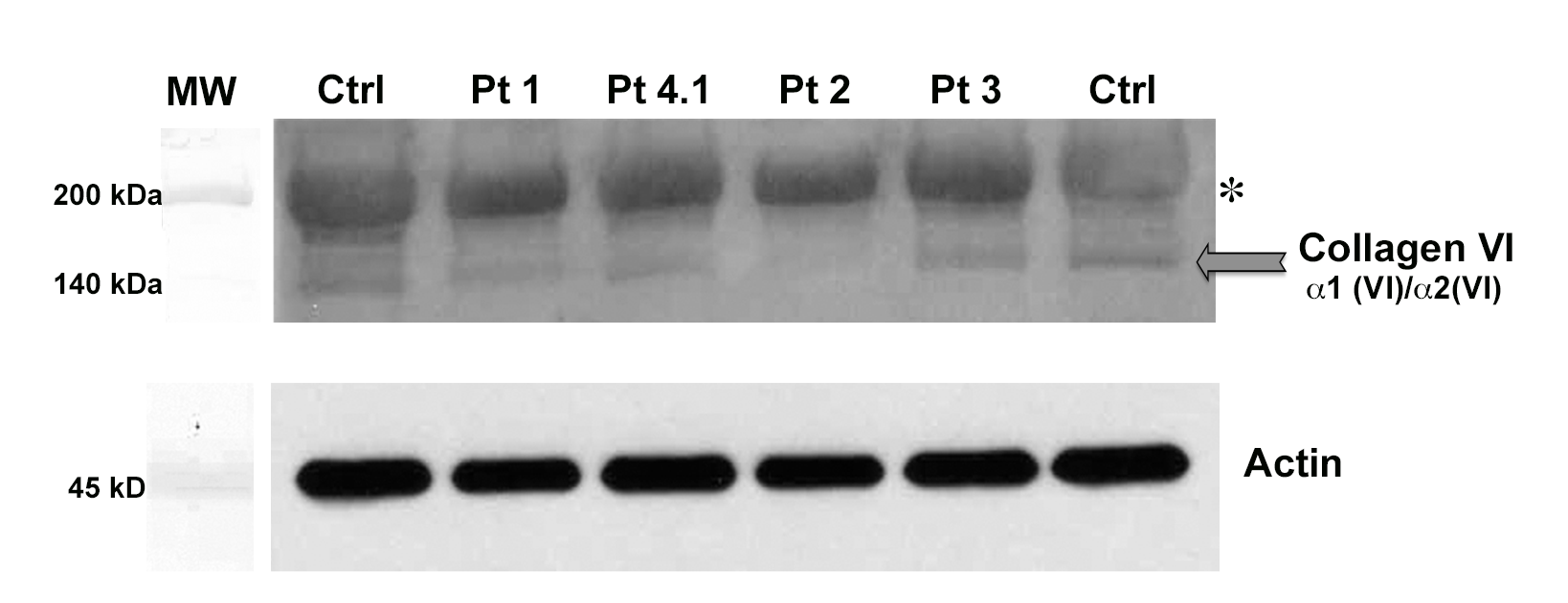

Supplement: Supplementary file 1 [file ijms-24-05551-s001.zip › Supplementary Figure S3.tif]
